# Polymeric nanoparticles—Promising carriers for cancer therapy

**DOI:** 10.3389/fbioe.2022.1024143

**Published:** 2022-10-07

**Authors:** Xiao Xiao, Fei Teng, Changkuo Shi, Junyu Chen, Shuqing Wu, Bao Wang, Xiang Meng, Aniekan Essiet Imeh, Wenliang Li

**Affiliations:** ^1^ School of Pharmacy, Jilin Medical University, Jilin, China; ^2^ School of Chemistry and Environmental Engineering, Changchun University of Science and Technology, Changchun, China; ^3^ School of Clinical Medicine, Jilin Medical University, Jilin, China; ^4^ Jilin Collaborative Innovation Center for Antibody Engineering, Jilin Medical University, Jilin, China

**Keywords:** polymeric nanoparticle, nanocarrier, cancer drug delivery, thermo-sensitive, pH-sensitive

## Abstract

Polymeric nanoparticles (NPs) play an important role in controlled cancer drug delivery. Anticancer drugs can be conjugated or encapsulated by polymeric nanocarriers, which are known as polymeric nanomedicine. Polymeric nanomedicine has shown its potential in providing sustained release of drugs with reduced cytotoxicity and modified tumor retention, but until now, few delivery systems loading drugs have been able to meet clinical demands, so more efforts are needed. This research reviews the current state of the cancer drug-loading system by exhibiting a series of published articles that highlight the novelty and functions from a variety of different architectures including micelles, liposomes, dendrimers, polymersomes, hydrogels, and metal–organic frameworks. These may contribute to the development of useful polymeric NPs to achieve different therapeutic purposes.

## 1 Introduction

It has been noticed for long that clinical application of many potent remedial drugs in cancer and other diseases have been limited due to the following reasons: 1) poor aqueous solubility, and consequently, minimal systemic bioavailability (Anitha et al., 2011; Tosca et al., 2021). During the treatment, patients need to be injected with a large amount of saline, which easily causes water intoxication (Lauersen and Birnbaum, 1975). 2) Instability *in vivo*—the drugs are dispersible in the whole living body and tend to be metabolized and eliminated, which causes the change of drug concentration *in vivo* and reduces its efficacy (Marczynski et al., 2021; Fontana et al., 2021). 3) Toxic side effects—the drugs are always toxic to the normal cells at the same time, including nephrotoxicity, neurovirulence, and gastrointestinal reactions (Chen Y. et al., 2020; Deng et al., 2021). Patients need to take cytoprotective agents, which increases their pain. So large doses are not acceptable at all, and the efficacy of the drug is limited. 4) Drug resistance—it is easy to cause multidrug resistance and reduce the effect of further treatment (Patil et al., 2010; Kim et al., 2013). Therefore, the development of new cancer drugs, the improvement of the absorption of the drugs, and the delivery of the drugs are urgent tasks.

Nanoparticles (NPs) are promising effective delivery tools as the candidates that overcome the difficulties in clinical applicability (Haley and Frenkel, 2008; Saw and Jon, 2020; Zheng et al., 2021). The colloidal particles with the size of 10–1,000 nm are termed as NPs. NPs can supply efficient therapeutic strategies by improving the bioavailability, stability, and target specificity to reduce side effects and overcome the limitations of conventional treatment methods (Gaumet et al., 2008; Yang et al., 2020; Ndebele et al., 2021). In addition, NPs have the potential to improve drug solubility, prolong cyclic half-life, and optimize drug pharmacokinetics (Verdurmen et al., 2021). NP-based drug delivery approaches have the potential of multifunction. They can disperse hydrophobic agents in aqueous media, thus improving the poor solubility, protecting the stability of drugs, and prolonging their circulation in the blood. They deliver the drugs to the target, therefore reducing the side effects and drug resistance. In general, NPs efficiently carry therapeutics to the target and are stable *in vivo* (Cotí et al., 2009; Sun et al., 2018; Li et al., 2020).

**FIGURE 1:**
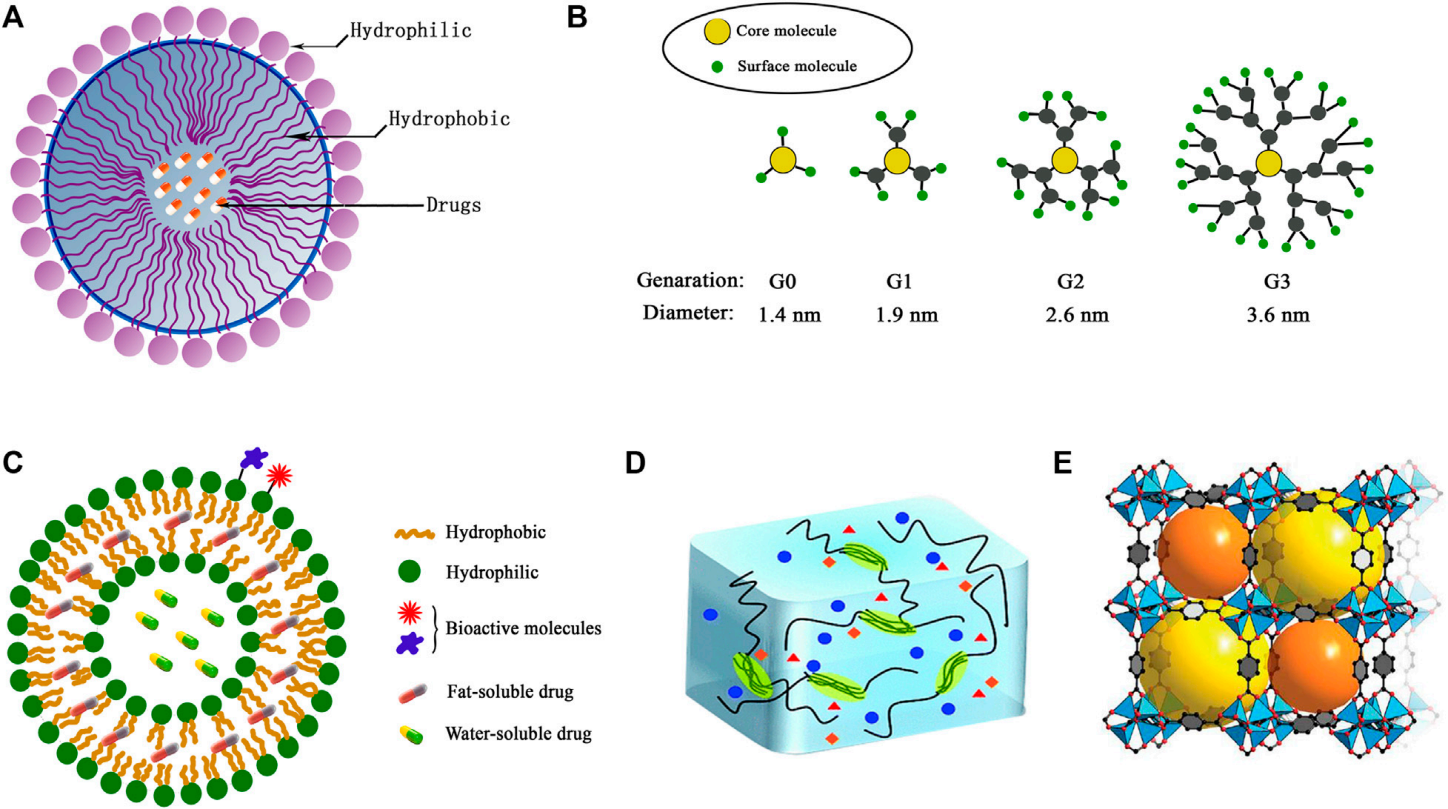
The structures of typical polymeric nanoparticles: **(A)** micelles, **(B)** dendrimers, **(C)** polymersomes, **(D)** hydrogel, **(E)** metal–organic framework (MOF).

Nowadays, drug-loaded NPs are particularly viable for efficient cancer therapy due to their improved drug delivery and therapeutic effects in various types of cancer (Wang et al., 2010; Rejinold et al., 2011; Truong et al., 2015). Polymeric nanocarriers with conjugated or encapsulated anticancer drugs, also known as polymeric nanomedicines, show a variety of different architectures including polymer–drug conjugates, micelles, nanospheres, nanogels, vesicles, and dendrimers (Alexis et al., 2010; Liu et al., 2014) (Figure 1). They attract more attention for their ability to co-deliver multiple therapeutic agents and to target the tumor cells (Liu et al., 2020). Broad classes of therapeutics including cytotoxic agents, small interference RNA (siRNA), chemosensitizer, antiangiogenic agents, and so on can be carried by the nanoparticle platforms (Hu et al., 2010; Liao et al., 2014; Lammers and Ferrari, 2020). NPs enhance their retention effect and permeability on the basis of the exclusive pathophysiology of cancerous cells. NPs accumulate in cells without being recognized by P-glycoprotein, which results in the increased intracellular concentration of drugs. NPs can deliver chemotherapies to tumor cells at the right time when needed. They take the drugs to the right place, at the right concentration with greater efficacy and reduced cytotoxicity in peripheral healthy tissues (Gaitanis and Staal, 2010; Ambasta et al., 2011; Shao et al., 2015). Therein, a targeting strategy as a multitargeting system (MTS) has attracted extensive attention (Purushottamachar et al., 2013). In this strategy, the surface of NPs is decorated with two or more functional ligands for recognizing different receptors on the cells, which can enhance the multivalent interactions between the NPs and cell surface, and help to improve cell recognition and internalization. In addition, there are other strategies including adding pH-sensitive groups to improve drug efficacy (Asad et al., 2021). As NPs modify the cancer treatment techniques, various multifunctional NPs are now being investigated. Following are the overview of some typical polymeric NPs.

## 2 Typical polymeric nanoparticle carriers

### 2.1 Micelles

The polymeric micelle delivery of anticancer drugs improve the tumor targeting of drugs and reduce the drug resistance, which has aroused extensive research interest in recent years (Chen J. J. Y. et al., 2020; Mignani et al., 2021; Son et al., 2021). The polymeric micelle consists of a hydrophobic core and hydrophilic shell. Their sizes vary in tens to hundreds of nanometers. Its hydrophobic core is capable of packaging various hydrophobic drugs, such as cisplatin, doxorubicin, camptothecin, paclitaxel, and so on (Wu Y. et al., 2010; Soltantabar et al., 2020). Most of the hydrophilic shells are made of polyethylene glycol (PEG). PEG helps micelles to escape recognition and capture by the reticuloendothelial system. So the drugs inside could stay in the bloodstream long enough. Through the enhanced permeability and retention (EPR) effect, drugs will be accumulated in tumor tissue passively (Hori et al., 2010; Liu T. J. et al., 2011; Zhao et al., 2020). In addition to these, by introducing small organic molecules, peptides, protein, sugar moieties, nucleic acid ligands (e.g., aptamer), antibodies, and other tumor-targeting groups to the surface of the micelles, or through the combination with magnetic nanoparticles, micelles may even target tumor proactively (Chen et al., 2013; Yu et al., 2014; Yu Z. et al., 2015). Therefore, the use of micelles as drug delivery can significantly increase the bioavailability and the anti-cancer ability of drugs such as chemotherapeutic compounds, DNA, and siRNA and at the same time, reduce their toxicity and side effects (Jiang et al., 2021). The first to prove the potential of clinic application of the micelles with active targeting capacity is the clinical study of PSMA-targeted polymeric nanoparticles (Hrkach et al., 2012). So far, various methods for anticancer drugs have been explored to enhance cell internalization and subcellular distribution. The basic standpoint is to discover the self-changes of physiological properties of tumors and therefore take advantage of these changes to improve the related structure of micelles and increase the anticancer efficacy.

#### 2.1.1 pH-responsive micelles

In previous decades, it had been found that the delivery of polymeric micelles for anticancer drugs responding to tumor acidity may be an effective way. Plenty of pH-responsive copolymers responsive to weakly acidic tumor microenvironments (pH: 6.5–7.0) have been explored (Wu X. L. et al., 2010; Yang et al., 2010). Tumors show different acidic environments, including the extracellular pH (pH_E_), and the environment is more acidic in the endosomal and lysosomal compartments of cancer cells (endosomes: pH 5.5–6.3 and lysosomes: pH 5.0–5.5) (Jiang et al., 2012). The average pH of normal tissue is around 7.4. Anoxia in the tumor cell prompts the anaerobic glycolysis to produce lactic acid. But the lack of a vascular system in tumor cells does not allow the lactic acid to be excreted completely, which leads to the acidic environment in tumor cells (Crayton and Tsourkas, 2011; Shen et al., 2012). The acidic environments in tumors can be treated as a signal to trigger the drug release of micelle carriers.

**FIGURE 2:**
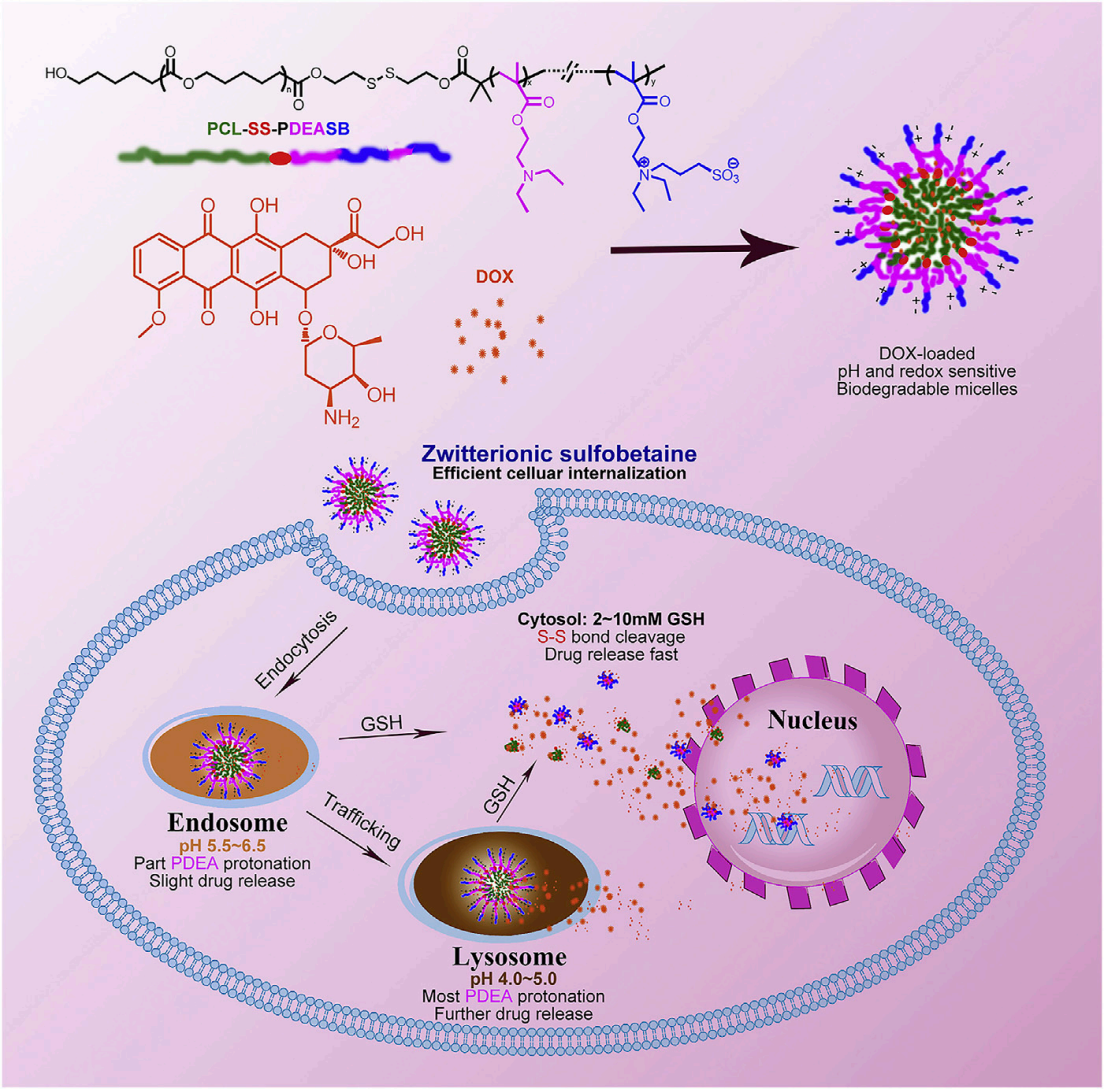
pH- and redox-sensitive micelles containing sulfobetaine for intracellular cell drug release. Adapted with permission from Cai et al. (2015).

pH-responsive polymer micelles are mainly prepared by self-assembly of amphiphilic blocks or grafted copolymers (Basak and Ghosh, 2013; Kim et al., 2014). pH-responsive polymer segments must be contained, which can be made by protonated or deprotonated polymers. A deprotonated polymer is alkalescent with amine, pyridine, imidazolyl, or other basic groups. The protonated polymer may contain a carboxylic acid group which shows weak acidity. When pH value varies, the ionization state of the copolymer changes, causing the difference of the solubility of micelles in water. Poly (N,N-diethylaminoethyl methacrylate) (PDEA), poly (4- or 2-vinylpyridine) (PVP), poly (L-histidine) (PHis), and poly (*β*-amino ester) (PbAE) are the most common alkalescence copolymers (Figure 2) (Vamvakaki et al., 2005; Manganiello et al., 2012; Cai et al., 2015). These coploymers are not soluble in neutral or alkaline circumstances. When they are in acidic environments, the basic group is going to be protonated and positively charged, which results in their solubility. The ones with carboxylic acid group are on the contrary. They are soluble in basic environments, such as the groups of poly (2-alkyl acrylic acid), poly acrylic acid, and so on. The pH of the transition state from insolubility to solubility (pH_T_) can be adjusted by the substituents (Gastaldi et al., 1996). The pH_T_ of pH-responsive polymer micelles suitable for the delivery of anticancer drugs should be 4.0–7.0 according to the acidic tumor environment (Xu et al., 2009). Another type of polymer used to prepare pH-responsive micelles is the acid-labile polymer. They can rapidly hydrolyze in a weakly acidic environment and complete the conversion from hydrophobicity to hydrophilia. To date, the chemical bonds rapidly hydrolyzing under acidic conditions are hydrazone, *β*-carboxamide, ester bonds, and so on (Agut et al., 2010; Lee et al., 2014; Shi et al., 2015). In general, pH-responsive micelles all have the ability to dissociate in tumor microenvironment and release the drugs.

**FIGURE 3:**
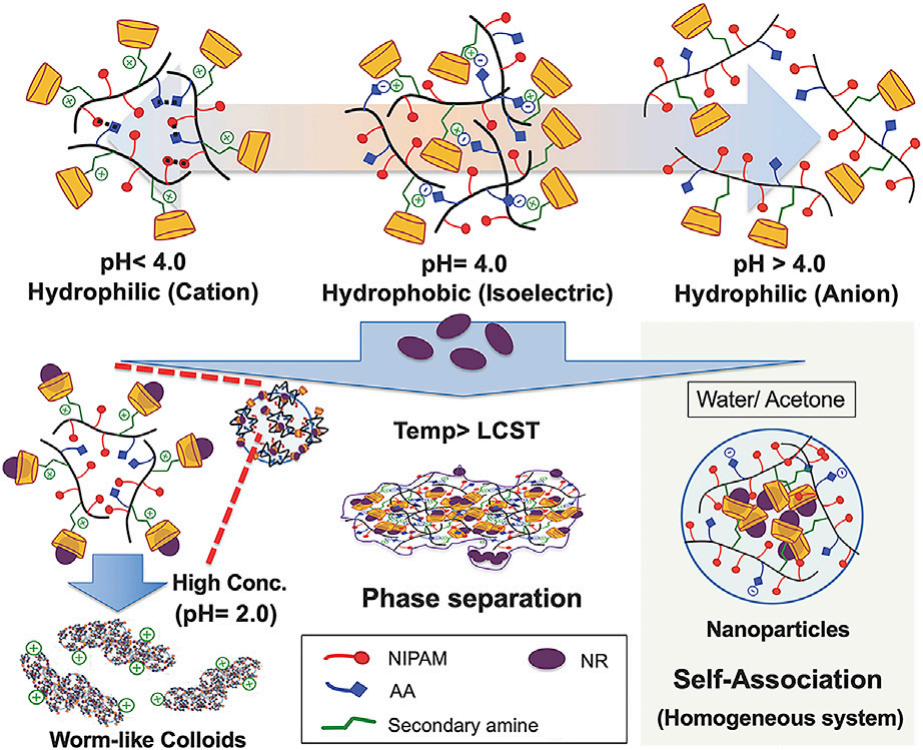
Colloidal assembly system comprising the poly (NIPAM-AA-β-CD) solution as a function of pH and temperature in both the homogeneous and inhomogeneous NR inclusion systems. Adapted with permission from Kawano et al. (2021).

People now focus more on the multifunctional pH-responsive micelles. Recently, a kind of micellar-like polymeric amphiphile modulated by host–guest interactions in the presence of a lipophilic dye to induce assembly and phase transition under precise pH control was studied (Figure 3) (Kawano et al., 2021). Jun Hu has reported a paclitaxel-loaded cationic micelle from a block copolymer of poly (L-histidine) (3.7 kDa) and shortly branched polyethyleneimine (1.8 kDa) (Hu et al., 2013). The micelle is cationic, and its surface can be shielded by a negatively charged complex mPEG (2 kDa)-block-polysulfadimethoxine (4 kDa) (mPEG-b-PSDM) at pH 7.4. That made the micelle charge shielded. It was proven by the experiment that at pH 7.4, two human cancer cell lines, MCF-7 and SKOV-3, uptake the unshielded and deshielded micelle rapidly, while the uptake of the shielded micelle was minimal. The *in vivo* results from a mouse model also showed significant anticancer therapeutic efficacy. Pengcheng Yu also reported a mitochondria-targeted pH-responsive micelle: it is DOX-loaded and pH-responsive (Yu P. et al., 2015). The micelles were synthesized by a pH-responsive diblock copolymer, poly (ethylene glycol)-block-poly (2-(diisopropylamino)ethyl methacrylate) (PEGb-PDPA), and a vitamin E derivate (D-a-tocopheryl polyethylene glycol 1000 succinate, TPGS). DOX was released by the acidic pH-triggered micelle dissociation. At the same time, the TPGS segment assisted the drug by targeting mitochondrial organelles and reduced the mitochondrial transmembrane potential. *In vitro* experiments on DOX-resistant MCF-7/ADR cells demonstrated the micelles reduced the IC_50_ of DOX by a sixfold magnitude. *In vivo* animal studies also showed its powerful efficacy. Many other pH-responsive micelles like these are still being explored. All these results imply that the pH-responsive micelles have significant potential for drug delivery.

#### 2.1.2 Reduction-responsive micelles

Reduction-responsive micelle is another effective delivery which has extensively been utilized for intracellular drug release as well. The point of reduction-responsive micelles is mainly based on the over-expressed glutathione (GSH) in cancer cells. GSH in the cytoplasm of tumor has a concentration of up to 10 mmol/L. Compared to normal tissues, the cancer cells exhibit an notably reduced microenvironment due to the higher concentration of GSH (Yan et al., 2012). For this kind of micelles, an important reduction-responsive bond–disulfide bond has to be mentioned, which generally cross-links the core and shell or acts as the linkage between PEG shell and micellar core (Xia et al., 2018). High intracellular GSH levels result in the break of this bond, and PEG (or the shell) detaches from micelles, which triggers drug release. A typical example with this function is the biocompatible reduction-responsive micellar system reported by Ding et al. (2013). It is made of methoxyl poly (ethylene glycol) (mPEG) and poly (3-benzyloxycarbonyl-L-lysine) (PZLL) with disulfide-link. DOX can be loaded by the micelles with loading efficiency of about 30 wt%. In phosphate-buffered saline with 10.0 mmol/L GSH, DOX-loaded micelle was accelerated to release DOX and show higher cellular proliferation inhibition toward HeLa and HepG2 cell lines. In addition to that, the water-soluble reduction-sensitive reversibly core-cross-linked micelles were also studied (Rapp and Deforest, 2021). It is based on poly (ethylene glycol)-b-poly (N-2-hydroxypropyl methacrylamide)-lipoic acid (PEG-b-PHPMA-LA) conjugates and can be triggered to release DOX in the presence of 10 mmol/L catalytic dithiothreitol (DTT) which provides the reduction environment.

#### 2.1.3 Photosensitive micelles

Photosensitive micelles mainly rely on the photosensitive segment. On exposure to light of appropriate wavelengths, the photosensitizers lead to the formation of reactive oxygen and therefore trigger the disruption of endosomal or lysosomal membranes (Ding et al., 2013; Xia et al., 2018; Rapp and Deforest, 2021). For instance, the polymeric micelles loading camptothecin (CPT) exhibited photosensitive endosomal escape, which reduced the cytotoxicity of camptothecin on photoirradiation (Nishiyama et al., 2009). Photosensitive platinum (IV) prodrugs (UVA-Pt2) are attached to polymers and self-assembled into micelles. The synthesized NP-UVA-Pt2 can be actively transported by tumor endocytosis, activated under UVA irradiation, and can release cisplatin to eliminate the tumor (Rong et al., 2014). Moreover, photosensitive platinum drugs can overcome the resistance of cisplatin (Hai et al., 2015).

**FIGURE 4:**
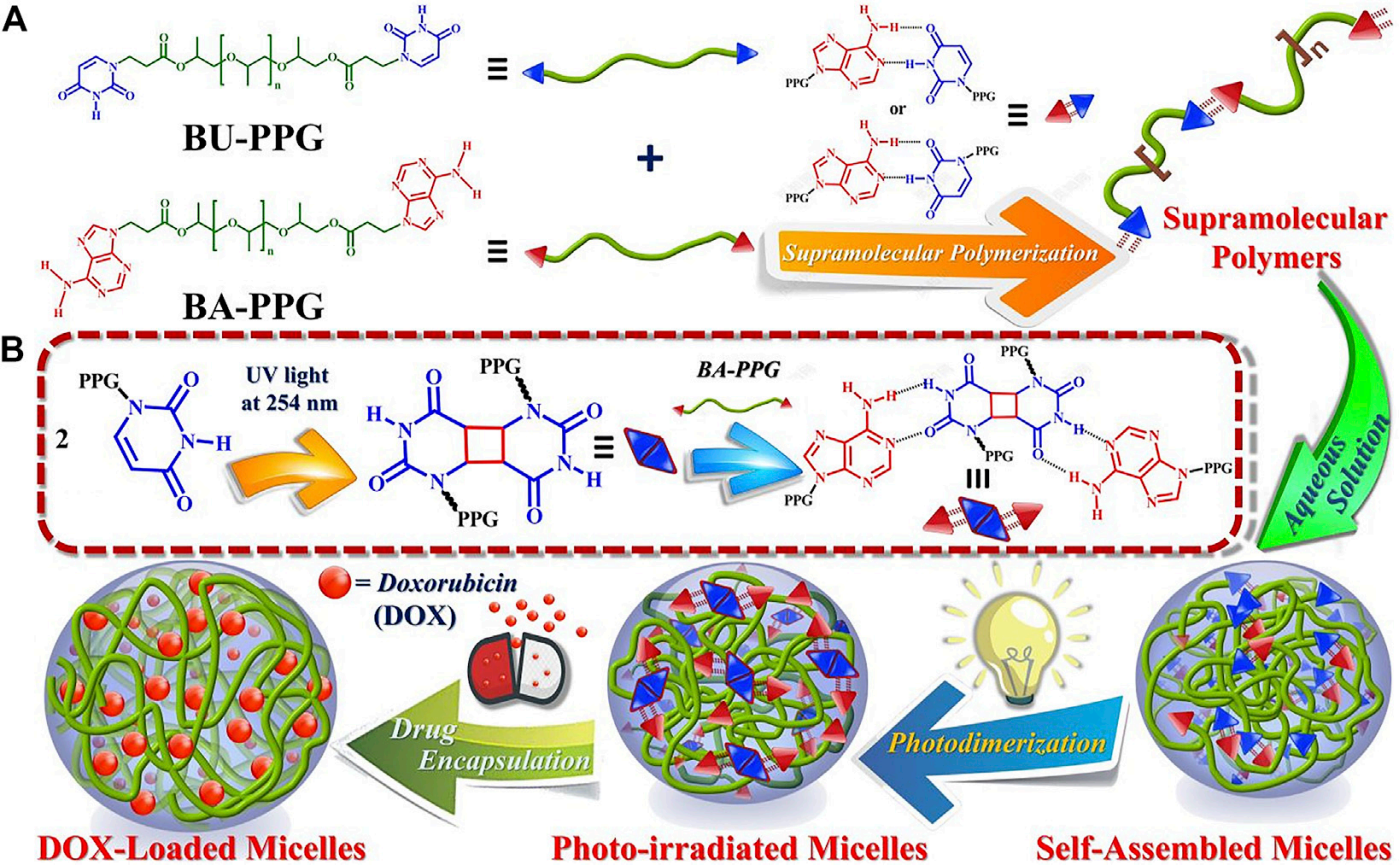
Schematic illustration of micelle formation and drug loading/release by photosensitive BA-PPG/BU-PPG in aqueous buffer solutions. Adapted with permission from Alemayehu et al. (2019).

The photosensitive supramolecular drug-loaded irradiated micelles had more potent cytotoxic effects against cancer cells exhibiting much higher cellular uptake efficiency (Figure 4) (Alemayehu et al., 2019). They rapidly entered the tumor cells to induce massive cell death. In addition to all the above, temperature-sensitive and enzyme-responsive micelles are also studied and show the potential to reach improved antitumor efficiency (Lee et al., 2007).

In general, the micelles can be used as carriers for the insoluble drugs which are with good therapeutic effect but have high toxicity. They are capable of loading insoluble anticancer drugs such as paclitaxel, hydroxycamptothecin, and doxorubicin with good results as we mentioned. They improve the solubility, the concentration, and the retention time of the drug at the tumor site, so as to improve the efficacy and reduce the side effects of the drugs. The micelles are also the carriers of gene drugs and protein drugs. The biodegradable micelle carriers have many advantages in gene delivery therapy, such as stability, non-toxicity, non-antigenicity, and gene protection. They act as non-viral vectors for plasmid DNA, small interfering RNA (siRNA), oligonucleotide, and so on. The development of safe delivery systems of the micelles will be the breakthrough of gene therapy. Furthermore, micelles also have applications in the traditional Chinese medicine. The micelles used in the study of active components, simple recipe, and compound recipe help to improve the dosage form and the therapeutic effect of the traditional Chinese medicine, which features in the modernization of traditional Chinese medicine. The group of Yan has obtained cationic nanomicelles as carriers of Chinese herbal medicine active components of ursolic acid. They enhance the drug efficacy in colorectal cancer treatment (Shu et al., 2020). At present, there are many researches on the multifunctional micelles. It is believed that with the developments of interdisciplinary research and new materials, nanomicelles will show more applications in pharmacy.

### 2.2 Dendrimers

A dendrimer is a synthetic nanomaterial. It was found and successfully synthesized by American chemist Tomalia D. A. in the early 1980s (Padias et al., 1987). In the past 20 years, dendrimers, with their unique structures and performance, have received attention in many fields such as material science and biomedicine (Liu X. et al., 2011; Li et al., 2016). The basic research and practical application of dendrimers have been studied continuously. With the development of the study on drug delivery, the delivery of dendrimers was explored. The dendrimers have been hot for a long time as the gene and drug delivery. Dendrimers have specific three-dimensional structures. It is the macromolecule which is synthesized nanometer by nanometer. Dendrimers can be distinguished from other polymers by its highly branched, tree-like structure. All branching monomers originate from one core, which can be divided into two types according to their growth directions: divergent growth and convergent growth. Different from ordinary polymers, dendrimers show low viscosity, high solubility, mixability, and high reactivity. At the same time, their volume and shape can be controlled in the synthesis process. The surface can be made into a dense area composed of different molecular groups, which play the role of hooks. In particular, each terminal functional group can be combined with the attachment point of polyvalent molecules on the surface of viruses and cells, so as to facilitate the adhesion of various useful molecules. A dendrimer parcels the drug in two ways. The drug molecules can be physically captured in the interior of the dendritic structure or covalently bonded to the surface or other groups of dendrimers forming polymer–drug conjugates. As drug or gene carriers, dendrimers selectively accumulate in the tumor tissue site actively by some targeted groups or passively through the EPR effect, which makes the delivery system targeted. It can be designed and synthesized for specific application. With these features, dendrimers are becoming a new type of promising cancer drug carrier. Dendrimers are safe and efficient non-biological carriers for the cancer drugs. In spite of these, there are still many problems to be solved, especially the specific toxicology and biocompatibility of dendrimers, which need to be further studied. It is believed that with the development of nanotechnology and the improvement of design and synthetic methods, dendrimers will eventually go into clinical application generally.

#### 2.2.1 Divergent growth dendrimer

**FIGURE 5:**
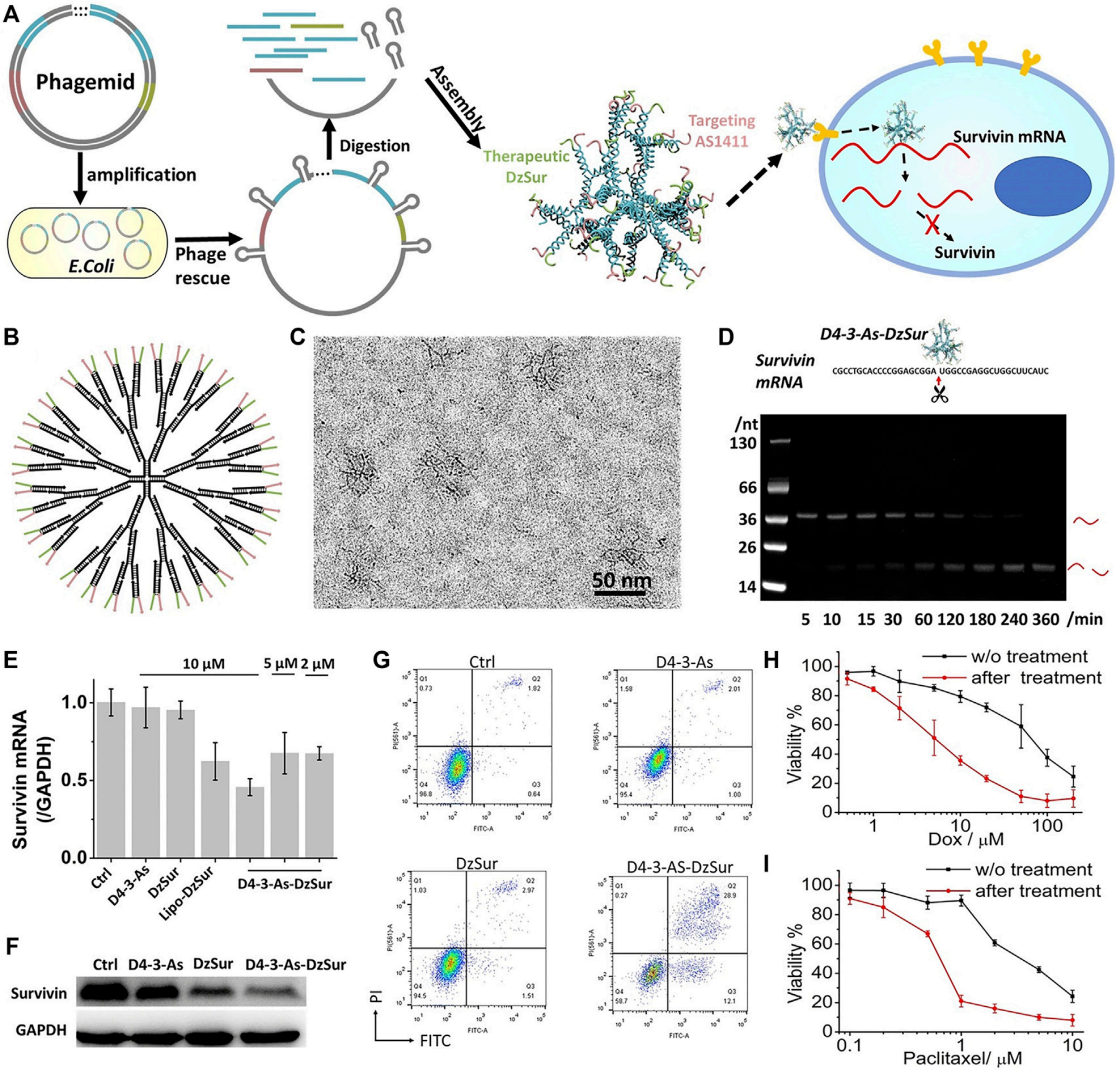
Targeted survivin regulation by the DNA-dendrimer-based nanomedicine D4‒3-As-DzSur. **(A)** Biotechnological production. **(B)** 2D structure. **(C)** Cryo-TEM image. **(D)** Survivin mRNA cleavage property. **(E)** qPCR tests. **(F)** Western blot analysis of survivin suppression in A549 cells. **(G)** Apoptosis analysis of A549 cell. **(H,I)** Chemosensitization effect on DOX **(H)** and paclitaxel **(I)**. (Lu et al., 2021).

The divergent growth method refers to the dendrimer growing and spreading from a initiator like ethanediamine or propylamine through gradual polymerization. Each polymerization reaction may form a new layer (or called generation) on the surface, marked as G_0_, G_1_, G_2_···G_10_. So the size and number of generations are decided by the synthesis procedure. For the steric hindrance effect formed by the compression of high-density branches, the self-growth will be limited. After the cyclic reaction, the generations can be up to 10 at most. The choice of initiator is very important, which determines the charge density of the whole polymer and the surface charge. The end functional groups are added at the end generation. The chemical properties of the molecules depend on the type of end-functional groups and their physical properties, such as solubility and viscosity, are also affected by the end groups. For example, the dendrimer-entrapped gold nanoparticles synthesized by Xiangyang Shi belong to this type. They are modified by folic acid (FA) and fluorescein isothiocyanate (FI) molecules which are water-soluble, stable, and biocompatible (Shi et al., 2007). FA and FI help the anti-cancer drug to exhibit bioactivity better. As the author reported, the FA- and FI-modified dendrimer-entrapped gold nanoparticles show high inhibition to kB cells (a human epithelial carcinoma cell line). PAMAM-G7 reported by Mitra Gholami was synthesized by the divergent growth method (Gholami et al., 2017), and a 6th generation dendrimer was synthesized in a single day by Per Antoni through the divergent growth method (Antoni et al., 2010). DNA-dendrimer-based nanomedicine constructed by the flexible 3-arm building blocks with a highly efficient one-pot DNA assembly was designed (Figure 5) (Lu et al., 2021). This is the first DNA nanomedicine (D4‒3-As-DzSur in literature 109) genetically encoded, biotechnologically produced, and directly self-assembled. The good targeted gene regulation has been proved by *in vitro* and *in vivo* tests.

#### 2.2.2 Convergent growth dendrimers

The convergent growth method is the second way to synthesize dendrimers. It refers to the dendrimer growing in reverse order as compared to the divergent growth method. It is triggered by the dendrons coupled with branch units. The dendrons target and link in one direction to the core molecule, and produce the tree-like polymer. The advantage of convergent growth method is that different terminal groups can be selected for different application requirements. The highly asymmetrical dendritic macromolecules containing well-defined functionalities at their periphery were synthesized by Craig J. Hawker using the convergent growth approach (Hawker and Fréchet, 1990). It was shown that the convergent growth method offered unparalleled control over the placement and number of functional groups at the periphery of dendritic macromolecules.

In summary, dendrimers achieved efficient gene transfer in a variety of cell tissues, which will lead to the targeted and multifunctional anticancer therapy. The dendrimers have many advantages such as no gene size limitation, being suitable for mass production with good reproducibility and low cost, and being easy to modify. They offer a new direction for the study of non-viral carriers. Different types of dendrimers have their own advantages and play a role in gene therapy, particularly the ones with the modifications of amino acids and peptides realize high transfection efficiency and targeting in specific cells. However, their biotoxicity and biocompatibility limit their development, so chemical modifications are needed. The modifications increase the uptake rate of cells, improve the efficiency of gene transfection *in vivo*, and expand the application of dendrimers. The modification of supramolecular parent material improves the membrane penetration of dendrimer, but the supramolecular parent material itself needs to be modified before binding to the dendrimer. Almost all the supramolecular parent materials, except natural cyclodextrin, were synthesized with complex steps, which limits their application. The modification from inorganic group confers dendrimers new properties and makes multifunctional gene vectors. However, inorganic substances cause the antigen and antibody reactions *in vivo*, which makes them unsuitable for safe gene therapy. Some functional responsive dendrimers improve the efficacy of drugs, while the clinical applications of them is still lacking. Therefore, the combination of organic and inorganic materials with dendrimers through physical or chemical methods is going to be explored to complete the safe multifunctional therapy of the dendrimers.

### 2.3 Polymersomes

Polymersomes are a kind of supramolecular aggregates formed by the self-assembly of amphiphilic molecules. They own spherical single-chamber or multi-chamber structures formed by closed bilayers, which are similar to the structure of cell membranes (Christian et al., 2009; Kim et al., 2009). As drug carriers, polymersomes modify the distribution of drugs in body, prevent drug degradation and inactivation, extend the action time of drugs, and reduce side effects. After being parceled by polymersomes, the drugs may prolong their circulation time in blood and target tumor selectively. The polymersomes are self-assembled by the synthetic or natural modified amphiphilic polymers (Pangburn et al., 2012). Compared with surfactants, liposomes, and other small vesicles, polymersomes show the advantages of easy molecular design, high intensity, good stability, strong permeability, and so on. In recent years, due to the rapid development of macromolecular self-assembly technology, as a new type of drug carrier, polymersomes have developed well and become the important current research on novel self-assembly drug delivery. Because the structure of polymersomes is similar to that of liposomes, they have a hydrophilic inner cavity and a hydrophobic membrane bilayer (Chandrawati and Caruso, 2012). So, similar to a liposome, it delivers hydrophilic drugs and hydrophobic drugs at the same time, being a unique drug carrier for these two kinds of active constituents. In addition, the hydrophilic shell on the surface of polymersomes shows better stability, enhanced permeability, and retention. Bioactive molecules, such as antibodies, polypeptides, and ligands, can be attached to the surface of polymersomes stably. These molecules also make the polymersome an active target or show other biological effects. Polymersomes can also be used as carriers for artificial oxygen (Wen et al., 2014). Different amphiphilic polymer molecules (different species, molecular weight, block proportion, polymer structure, etc.) affect the physical and chemical characteristics (particle size, film thickness, permeability, drug loading, etc.) and the *in vivo* behavior of the polymersome, which makes the polymersome system more controllable. Therefore, polymersome, as a new type multifunctional drug delivery, because of its high drug loading, high encapsulation efficiency, stability, and controllability, can be used to improve the targeting delivery and bioavailability of anti-cancer drugs. Like their low molecular weight analogues liposomes, they can be stimuli-sensitive (Li and Kellera, 2009; Siegel, 2014). Stimuli include pH, temperature hydrolysis, oxidation, reduction, light, and so on. Here, we discuss the thermo- and pH-sensitive polymersomes.

#### 2.3.1 Thermo-sensitive polymersomes

For the polymers sensitive to temperature stimulation, their hydrophobicity increases as the temperature changes, and the polymersomes are formed so. The hydrophobic segment most commonly used for thermo-sensitive polymer is poly (N-isopropylacrylamide) (PNIPAAm) (Lorenzo et al., 2005; Cao et al., 2007). PNIPAAm has lower critical solution temperature (LCST) of 30–50°C. It is completely soluble in aqueous solution when the temperature is lower than LCST, while it becomes insoluble when the temperature is higher than LCST. The polymers with PNIPAAm block self-assembly to form micelles or polymersomes when they are more hydrophobic. Drugs can be loaded in these polymersomes at temperatures below LCST. When the temperature is above LCST, these polymersomes are collapsed to release the drugs. The drug release responding to the temperature change makes thermo-sensitive polymersomes, and they become excellent carriers for controlled drug release applications. So far, the blocks have been used for thermo-sensitive polymersomes including poly (N-(3-aminopropy1)-methacrylamide hydrochloride)-b-PNIPAAm, poly (2-einnamoylethyl methacrylate)-b-PNIPAAm, PEG-PNIPAAm, and so on (Pamies et al., 2009; Zhang et al., 2011; Quan et al., 2013). Thermo-sensitive poly (N-isopropylacrylamide-co-acrylamide- coallylamine) (NIPA-AAm-AH) polymersomes reported by Maham Rahimi with lower LCST can be incorporated with various molecules at the surface (Rahimi et al., 2008). The polymersomes were synthesized by the approach of free-radical polymerization. They can release the DOX most at 41°C. Biao Yang prepared the pore-covering polymer brushes by PNIPAAm onto the PET (Yang and Yang, 2003). With the change of the temperature, the pore size varied with the swelling and shrinkage of poly-PNIPAAm brushes and the membranes’ fluxes were adjusted.

#### 2.3.2 pH-sensitive polymersomes

pH-sensitive polymers have titratable functional groups and can be protonated or deprotonated by adjusting environmental pH value. Thus, the solubility of the polymer will be changed to prepare pH-sensitive polymersomes. The pH-response can be obtained either by polyacid blocks which lose H^+^ and become ionized by a higher pH variation, or by polybase blocks that can be protonated and insoluble at lower pH. The best advantage of pH-triggered release is the fast response of the system. The release occurs almost instantaneously. For example, in the case of poly (2-(methacryloyloxy)ethyl phosphorylcholine)-b-poly (2-(diisoprop ylamino) ethylm ethacrylate (PMPC-PDPA) polymersomes reported by Du Jian-zhong, when the pH value of the solution altered from 2 to greater than 6, the hydrophobicity of PDPA was enhanced due to the deprotonation of the tertiary amine group in the PDPA segment, and the di-block copolymer PMPC-PDPA formed polymersomes (Du et al., 2005). In a similar way, for the di-block copolymer poly (butadiene)-b-poly (L-glutamic acid) (PBD-PGA), the particle size of 100–150 nm can be prepared through deprotonation of PGA by adjusting pH value. Polymersomes composed of poly (ethylene glycol)-polyester reported by Fariyal Ahmed were shown to break down into membranelytic micelles within hours at low pH (Ahmed et al., 2006). The antitumor drug with their delivery kill growing tumor rapidly and effectively. Thermo- and pH-sensitive polymers synthesized by N-isopropylacrylamide and methacrylate monomers derived from cholic acid with ethylene glycol and oligo spacers through free-radical polymerization were also reported by Benrebouh et al. (2001).

The polymersomes have so many advantages as we mentioned and they can deliver the water-soluble and hydrophobic drug molecules at the same time. They are widely used to encapsulate vulnerable proteins and genetic drugs. The time in blood circulation of the polymersomes can be extended with PEG modification. Drug release can be regulated by changing the type of polymer and the proportion of hydrophilic and hydrophobic segments. Stimulus-responsive polymersomes avoid unnecessary drug leakage, improve bioavailability, and reduce side effects. Targeted polymersomes enable targeted drug delivery. Compared with liposomes and other traditional NP delivery systems, polymersomes are easier to be prepared using cheaper raw materials and with better stability, which has become a new predominant NP delivery system. However, the clinical application of polymersomes still faces some challenges. First of all, the sterilization of polymersomes is the main obstacle to be used as nano-carriers. Because the sterilization temperature is higher than the transition temperature of gel liquid in the polymersome preparation, heat energy will break their structures, leading to drug leakage from the polymersomes, so heat sterilization (such as dry heat sterilization and high pressure steam sterilization) is not suitable for the polymersomes. Membrane hyperfiltration is not suitable for polymersomes with particle diameters larger than the membrane aperture (0.22 μm) either. The preparation of polymersomes under aseptic conditions may be a solution. Many pharmaceutical preparations, such as eye ointment, eye drops, and injections, are sterilized by gamma ray, which produces less heat. Therefore, gamma-ray sterilization has the potential to be applied to the sterilization of polymersomes. In addition, the potential toxicity is another limitation for the clinical application of polymersomes. The biodegradability of polymersomes, their responses to stimuli, and targeting ability need further study. Meanwhile, the mechanisms of circulation, distribution, and metabolism of polymersomes in the body are not very clear. Solving these problems will be an important task for researchers.

### 2.4 Hydrogels

Under certain conditions, colloidal particles connect with each other in the dispersion medium to form a spatial network. Meanwhile, the dispersion medium is filled in the interspace of the structure. The dispersion system formed thereby is called hydrogel. The hydrogel contains large amount of liquid without mobility. Their affinity to imbibe water is attributed to the hydrophilic groups such as -OH, -CONH-, -CONH_2_-, and -SO_3_H in the structures (Hamidi et al., 2008). So hydrogels are described as three-dimensional configurations capable of absorbing large amounts of water or biological fluids. Hydrogels composed of 3D hydrophilic polymer chains have unique configurations and adjustable physicochemical properties (Peng et al., 2020). Even though the hydrogels absorb water well, they cannot be dissolved in an aqueous solution. When in aqueous surrounding environment, hydrogels will swell as a consequence of the critical crosslinks in the structure. These crosslinks can be physically (entanglements or crystallites) or chemically (tie-points and junctions) formed by the present of covalent bonds, hydrogen bonds, van der Waals interactions, or physical entanglements (Bernhard and Tibbitt, 2021; Zhang et al., 2021). The hydrogels can be generally divided into two groups: elastic hydrogel and brittle hydrogel. Elastic hydrogel is permeable. With the loss of the dispersion medium, the volume of elastic hydrogel will obviously decrease, and when the dispersion medium is reabsorbed, the volume increases, such as gelatin. The shape and volume of brittle hydrogel, such as silica gel, do not change when the dispersion medium is released and absorbed. Because of the small particle size, high water content, long retention time in blood, and degradability, hydrogel can be used as a carrier for cancer drugs. However, nanogels are mostly prepared in traditional ways. The release process is difficult to control, and some cross-linking agents are toxic to the body. In order to improve the safety and efficiency of hydrogel carriers in clinical drug delivery, new materials and cross-linking agents are synthesized to obtain multifunctional nanogels with high drug load, strong controllability, and good safety, so as to benefit from its advantages in drug delivery. Controlled-release or stimuli-induced-release hydrogel systems have become the focus of attention.

#### 2.4.1 Temperature-sensitive hydrogels

Due to the special constituents, the hydrogels repeat swelling-deswelling conversion. Temperature-sensitive hydrogels, also known as thermo-responsive hydrogels, are the ones accomplishing the conversion by responding to the environmental temperature changes. Most of the temperature-sensitive hydrogels have hydrophobic groups, such as methyl, ethyl, and propyl groups. As we mentioned, among the polymersomes, PNIPAAm is probably the most widely used. The hydrogels made up of LCST polymers display shrinkage when the temperature rises above the LCST. At lower temperatures, the hydrogen bonds in the hydrophilic block of polymer and water dominate to increase the dissolution in water. While with the temperature increases, hydrophobic interactions in hydrophobic segments dominate, and hydrogen bonding becomes weaker. That results in the hydrogel net shrinking because of interpolymer chain association through hydrophobic interactions. The more the hydrophobic constituents are contained, the lower the LCST becomes. LCST could be controlled by the ratio of hydrophilic and hydrophobic segments. The hydrogels with PNIPAAm show faster shrinkage and sensitivity to additional stimuli. For example, the temperature-sensitive hydrogels based on the interpenetrating network of PNIPAAm was immobilized on porous silica gel and hydroxyapatite (Shin et al., 2001; Shin et al., 2002). The ones with chitosan and hyaluronic acid were studied with their physicochemical characteristics. *In vitro* drug release and *in vivo* pharmacodynamics were examined in detail (Ayon et al., 2015). The ones with loaded carboplatin for controlled drug delivery have also been explored (Andre et al., 2007). These series of studies on the temperature-sensitive hydrogels in the pharmaceutical field showed promising results. Recent studies on temperature-sensitive hydrogels have shown that hydrogen bonding and hydrophilicity of polymers are important factors affecting the quality of delivery. At the same time, the large concentration and the addition of non-water-soluble agents will bring up safety problems. So modifying its structure or being mixed with other auxiliary materials or environmentally sensitive materials are the effective ways to improve the response to temperature, so as to prolong the drug retention time and improve the efficiency.

#### 2.4.2 pH-sensitive hydrogels

pH-sensitive hydrogels have been most frequently used to develop controlled release systems. pH-sensitive hydrogels generally contain ionic groups—acidic or basic groups that either give out or accept protons in response to environmental pH changes. The change of the pH value will affect the electric charge of ionic groups of the hydrogels, which leads to the increase of charge repulsion, thus causing the hydrogel to change from collapsing state to stretching state. That is the process of swelling and deswelling. The microenvironment pH of tumor cells is more acidic than the normal ones, so the tumor cell can be the trigger of drug release of the pH-sensitive hydrogels. The hydrogels dissolve more or swell more at low pH due to ionization. Different comonomers give different hydrophobicity to the hydrogels, causing different pH-sensitive behaviors. Examples of these are hydrogels based on poly (lactic acid), methoxyl poly (ethylene glycol), and itaconic acid prepared by Ke Wang; pH-responsive co-polymeric hydrogels studied by Umaira Rehman; and polyacrylamide grafted pectin pH-sensitive hydrogel reported by Prashant B. Sutar (Omidia et al., 2019; Sarfraz et al., 2019; Rehman et al., 2021).

#### 2.4.3 Other stimuli-sensitive hydrogels

**FIGURE 6:**
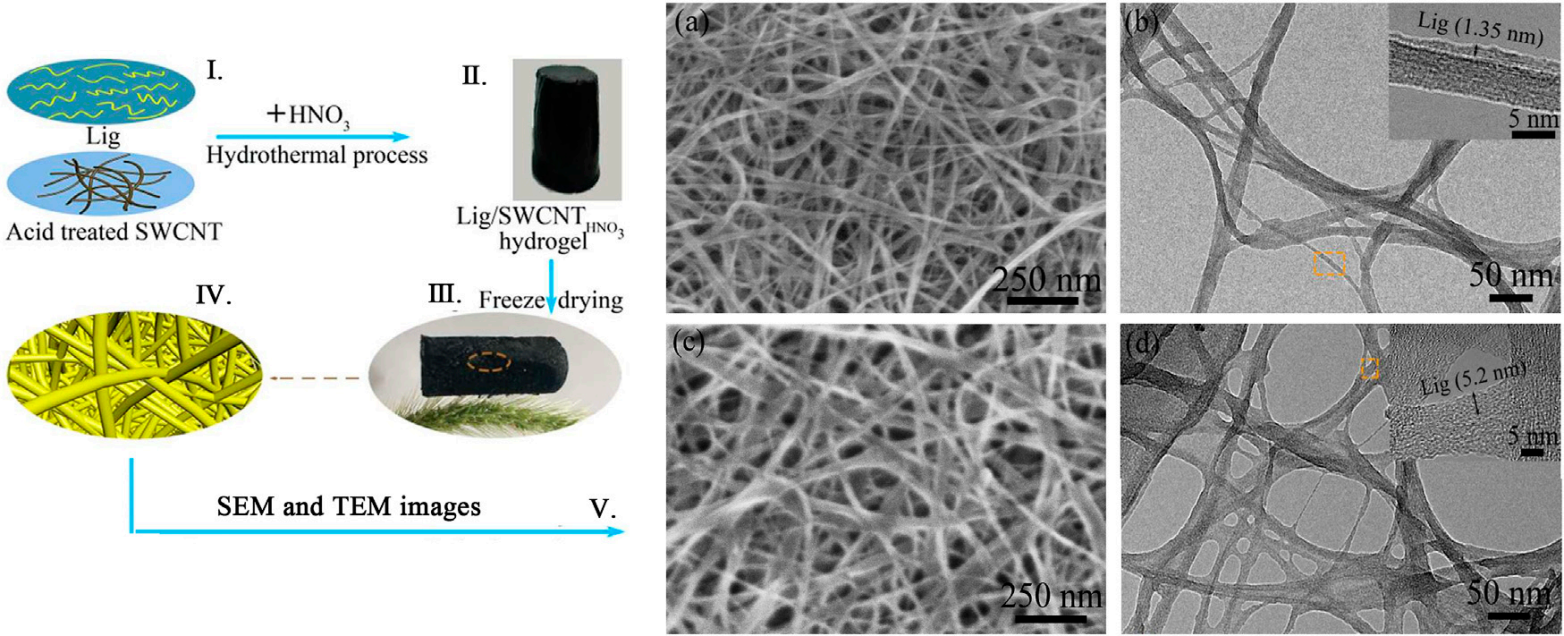
(Ⅰ–Ⅳ) Aerogel assembled by Lig (lignosulfonate) and acid treated SWNCT (single-walled carbon nanotubes). (Ⅴ) SEM and TEM images of Lig/SWCNT and Lig/SWCNT_HNO3_. Adapted with permission from Peng et al. (2018).

Electro-sensitive hydrogels shrink or swell in the presence of an applied extra electric field. In some cases, the hydrogels show swelling on one side and deswelling on the other side, resulting in the hydrogels bending. The hydrogel shape changes (swelling, shrinking, and bending) with the trigger of the electric field. That is the basis of the electro-sensitive hydrogels, such as carbon nanotube-coated hydrogel hybrids made by Servant et al. (2013). The visible light-sensitive hydrogel is readily available, low-priced, safe, clean, and easily manipulated. They are prepared by introducing a light-sensitive chromophore to the hydrogels, such as the polypeptide hydrogel synthesized by Charati et al. (2010). In addition to these, pressure-sensitive, specific ion-sensitive, and antigen-responsive hydrogels were all studied (Ikeda et al., 2010; Liu et al., 2013; Peng et al., 2018). The dibenzo-18-crown-6 copolymer was introduced into the PNIPAM system, which showed ionic sensitivity due to selective complexation with the crown ether unit, making the hydrogel show good ionic response to potassium (Figure 6) (Peng et al., 2018).

Hydrogels have been widely used in drug delivery due to their excellent biocompatibility and high water content. They can release drugs selectively and continuously respond to different environments. Related products especially the transdermal hydrogels are already on the market. The dosage forms are diverse and show different advantages such as high drug loading efficiency, good permeability, and high bioavailability. Hydrogels control drug release more accurately, significantly improve the solubility of some drugs, and they are easy to remove. At present, hydrogel is still a hot topic in academic research. As can be seen from the launch of the hydrogel products, hydrogel has broad application and market prospect. However, some problems still restrict the application of hydrogel in drug delivery. For example: 1) most of the hydrogels have relatively low tensile strength and elasticity, which makes them easy to break; 2) water swelling occurs and the swelling rate is high; and 3) hydrogels do not degrade easily and lack biological functions. Therefore, there are two directions for the future research of hydrogels. One is the structural transformation of existing hydrogel materials. The other is exploring new hydrogels with better performance, and designing a stimulation-responsive hydrogel drug delivery system.

### 2.5 Metal–organic frameworks

**FIGURE 7:**
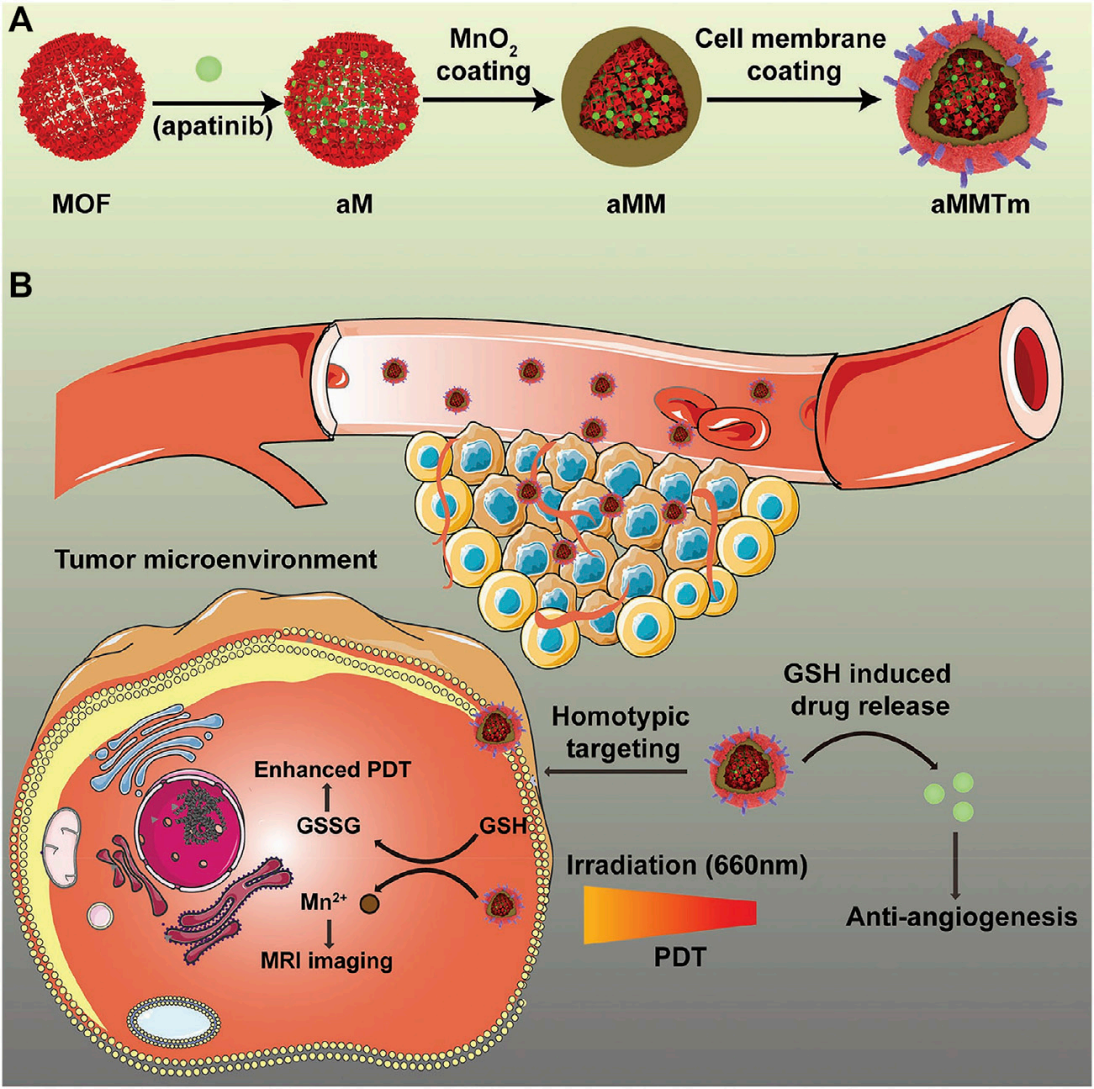
Schematic illustration of an integrated nanosystem apatinib-loaded MOFs (aMMTm) preparation and proposed combination therapy of photodynamic therapy and antiangiogenesis. Adapted with permission from Min et al. (2019). **(A)** The construction of porphyrinic metal‐organic framework nanoparticle aMMTm through drug loading, MnO_2_ coating and tumor cell membrane decoration. **(B)** Proposed action mechanism of aMMTm in a mouse tumor model. The tumor cell membrane camouflage may help the nanoparticles to escape early clearance and to target tumor tissue through homotypic affinity.

Metal–organic frameworks (MOFs) refer to the infinite network of crystalline materials formed by the self-assembly of transition metal ions or a metal cluster with the organic ligand. They are mainly used in the field of chemistry research such as magnetism, optics, and biological imaging (Chen G. et al., 2020; Chang et al., 2021). In recent years, medical researchers have reduced the size of such materials to the nanometer scale (NMOFs) and tried to use them in the fields of biological medicine storage, slow release, biological imaging, and the transmission of gaseous signal molecules (Liang et al., 2016; Wang et al., 2020). NMOFs can be synthesized by reprecipitation, reverse microemulsion, solvothermal method, or template synthesis method. NMOF has a large specific surface area, which makes it possible to be used as a drug carrier. NMOF shows good polymorphism and tunability due to the diverse types of the metals and the various coordinating abilities of the ligands. The coordination numbers of different metal ions are different, and the same metal ion may have different coordination numbers in different coordination environments. There are numerous organic ligands, especially those containing multiple carboxyl groups. Therein, the carboxyl group has a variety of coordination modes, and there may be two or more carboxyl groups in the ligand, so the coordination mode is very complicated. These all increase the adjustability of NMOFs. The structure of NMOFs is also affected by the synthesis condition. So, numerous various kinds of NMOFs are obtained by now, such as square shape, brick wall shape, diamond shape, quartz shape, ladder shape, lattice structure, and so on (Tavra et al., 2015; Chen J. et al., 2020; Lazaro et al., 2020). Storage and the release of drugs in the porous material are determined by a series of factors, including the pore size, pore morphology, pore-to-pore connectivity, and guest affinity. For traditional porous materials such as silicon, the capacity is not high enough, so loaded drugs are difficult to release. Porous NMOFs with large volume of aperture and regular structure can achieve high drug loadings and controlled drug release. The reported NMOFs as carriers have shown high drug loading. They load nonsteroidal anti-inflammatory drugs, antiviral drugs, and the antitumor drugs. The active exchange of NMOFs was completed almost exclusively through clathrin-mediated endocytosis, while the uptake was concerned with both clathrin and caveolae-mediated endocytosis. The NMOF is situated mostly in lysosomes against lysosomal degradation for further degradation in the cytosol, and drugs are released and distributed to different cellular organelles (Figure 7) (Min et al., 2019). The advantage of the NMOF is that it can be administered through a variety of ways and improve the pharmacokinetics of loaded drugs. Experiments showed it improves the antitumor activity of the controlled release system applied to the drugs based on Pt. Moreover, Maspoch etc. chose doxorubicin, camptothecin, and several other anticancer drugs to be loaded by MOFs through the self-assembly process (Imaz et al., 2010). Because these drugs have fluorescent properties, the release process was monitored. The NMOF as a drug delivery carrier greatly reduces side effects for the body and improves the drug efficacy in the focal area of body. Through chemical modification, NMOF forms a new drug release system and has become a hot research topic.

#### 2.5.1 Ordinary metal–organic frameworks

Here, the ordinary MOFs refer to the ones that are not environmentally sensitive. Through the modification to make them carry a variety of functional groups, MOFs for cancer drug delivery show different characteristics. Such as the NMOFs modified by folic acid, through the mutual connections of organic groups, the surface is filled with folic acid, which make the NMOF a folate receptor with targeting property (Sun et al., 2012). 5-FU uracil was loaded in, and the release could be sustained. This NMOF with larger drug loading capacity reduced the side effects and overcame the disadvantages. It has application prospects in antitumor treatment. The group of Lin synthesized Fe(III)-carboxylate nanoscale metal–organic frameworks with high specific surface area by the rapid microwave method (Rieter et al., 2008; Pashow et al., 2009). The ferric center is Fe^3+^ ion. 1,3,5,7-tetramethyl-4,4-difluoro-8-bromomethyl-4-bora-3a,4a-diaza-s-indaeene (Br-BODIPY) and ethoxysueeinato-cisplatin (ES-CP) were used to increase the histocompatibility. Coated with a shell of SiO_2_ with Na_2_SiO_3_ as the silicon source, the MOF was bonded with fluorescence and anticancer drug through a modified covalent bond. The drug loaded in the ferric MOF showed biotoxicity similar with cisplatin to human colon cancer cell line HT-29 at the same concentration. The nanoscale MOFs for the co-delivery of cisplatin and pooled siRNAs was also studied by Lin (Kathryn et al., 2009; He et al., 2014). The MOFs enhanced therapeutic efficacy in drug-resistant ovarian cancer cells.

#### 2.5.2 Stimuli-sensitive metal–organic frameworks

**FIGURE 8:**
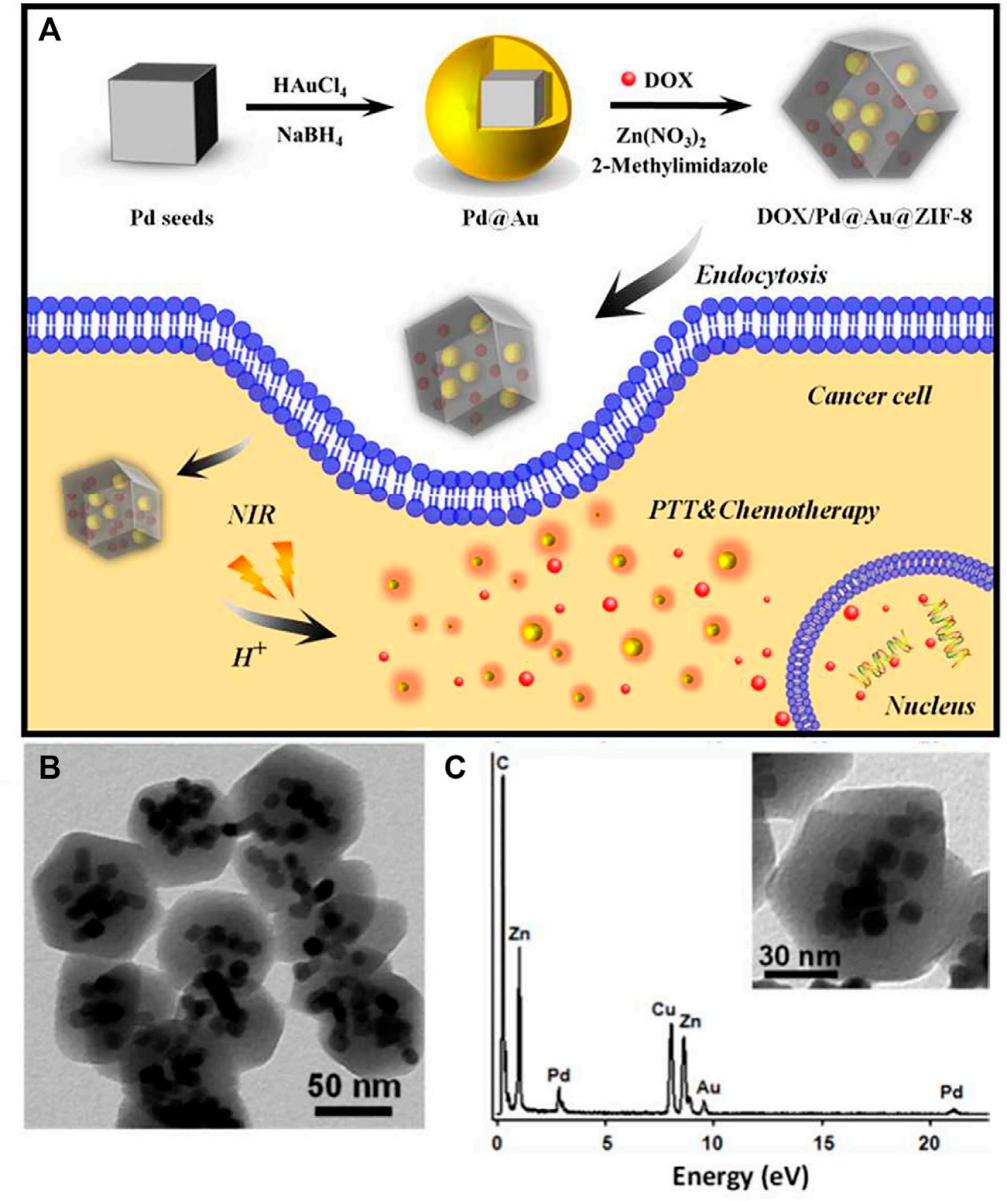
**(A)** DOX/Pd@Au@ZIF-8 and its application in pH and near-infrared-triggered chemotherapy photothermal synergistic treatment of cancer cells; **(B)** DOX/Pd@Au@ZIF-8 TEM images; **(C)** DOX/Pd@Au@ZIF-8 TEM-associated EDX spectra. Adapted with permission from Yang et al. (2017).

Compared with other drug deliveries, the study of MOFs started late and is under development. Even though environment-sensitive MOFs are at the early stage, various stimuli-sensitive MOFs have been synthesized. A class of thermo- and solvatochromic metal–organic frameworks based on 4-(pyridin-4-yl)benzoic acid reported by Gift Mehlana was synthesized by solvothermal methods (Mehlana et al., 2012). pH-sensitive MOF with co-encapsulation of Pd@Au nanoparticles and doxorubicin for pH- and NIR-triggered synergistic chemo-photothermal treatment of cancer cells showed an excellent synergistic treatment effect on SMMC-7721 cells even at low concentrations (Figure 8) (Yang et al., 2017). Other pH-sensitive MOF like hollow mesoporous silica (HMS)@MOF capsules for DOX showed much higher cytotoxicity than the free drugs (Yang et al., 2017). Additionally, a core@shell nanocomposite material with biocompatible bovine serum albumin (BSA) as the core and pH-sensitive MOF as the shell loading DOX was prepared (Liang et al., 2018). It released DOX at low pH (5.0–6.0) with higher efficacy against the breast cancer cell line MCF-7. These all accelerate the development of MOFs for anticancer drug deliveries.

As a new type of organic-inorganic hybrid crystalline porous material, MOF has the advantages of large specific surface area, high porosity, and diverse structures. However, there are also some problems to be solved in the application of MOFs: 1) The present preparation technology for most MOFs is not suitable for industrial production. It requires high precision of the instrument, which is still mostly confined to laboratory preparation. To overcome it, we need to make more efforts in the production process and instrument specifications. 2) There is some toxicity of the MOFs. The preparation of MOFs mostly relies on organic solvents as the medium, resulting in the residue of organic solvents in the pores, causing toxicity. However, the selectivity and synthesis efficiency of organic ligands will be affected if water is used as the synthetic medium due to the poor solubility. At the same time, the surface tension of water is large, and in the activation process before drug loading, the stress on the pores will be generated with the removal of water, leading to the pore collapse and the reduction of drug loading. This can be improved by the optimization of the solvent and activation process. A good synthetic medium should be non-toxic, volatile, and with low surface tension. The freeze-drying method and supercritical CO_2_ fluid can protect the pore structure of MOFs by the sublimation process and less stress of supercritical CO_2_ fluid on pore, so as to improve drug loading efficiency. More methods will be studied. 3) At the beginning of the development, many MOFs are used for adsorption, catalysis and so on. The organic ligands are chosen according to the chemical properties, which is inevitably irritating to the human body. From the medicinal point of view, researchers should pay attention to the optimization of their organic ligands. The use of ligands with specific functions or anticancer pharmacological effects can further improve the anti-tumor effect, and shows the advantage of combining drugs with adjuvants. 4) Different MOFs may have different stability and safety. There are many configurations and types of MOFs. However, few studies evaluated the metabolic process, safety, or biocompatibility of different types of MOFs *in vivo* and *in vitro*, so specific studies are necessary in this field to provide sufficient evidence of the safety and support the clinical application of MOFs. Interdisciplinary cooperation of chemistry, materials, and biomedicine is deepening. The research is expected to further develop MOF materials in biomedicine. The materials, cargo and *in vivo*/*vitro* test status of some typical nanoparticles mentioned above are summarized in Table 1.

**TABLE 1:** The materials, cargo and *in vivo*/vitro test status of some typical nanoparticles.

Type	Function	Materials	Cargo (not limited to this)	*In vivo* test	*In vitro* test	References
Micelles	pH-responsive	AP peptide/PEG-poly_(D,L-lactic acid)_/(ethylene glycol) (MPEG)-poly (β-amino ester	Doxorubicin	Done	Done	Wu X. L. et al. (2010)
		Hyperbranched aliphatic polyester, Boltorn H40/poly (ɛ-Caprolactone/poly (malic acid) (PMA-co-PCL)/poly (ethylene glycol)/folate	Doxorubicin	-	Done	Yang et al. (2010)
		Polymethacrylates (PMA)-grafted poly (amidoamine) (PAMAM)/folate-PEGylation	Paclitaxel	Done	Done	Shen et al. (2012)
		Poly (ɛ-caprolactone)-SS-poly (N,N-diethylaminoethyl methacrylate)-r-poly (N-(3-sulfopropyl)-N-methacrylate-N,N-diethylammonium-betaine) (PCL-SS-PDEASB)	Doxorubicin	-	Done	Cai et al. (2015)
	Reduction-responsive	Hyaluronic acid-tocopherol succinate (HA-ss-TOS) polymers/hyaluronic acid-tocopherol succinate (HA-TOS)	Paclitaxe	Done	Done	Xia et al. (2018)
		Poly (L-histidine)-block-short branched polyethyleneimine (PHis-b-sbPEI)/mPEG-block-polysulfadimethoxine (mPEG-b-PSDM),	Doxorubicin	-	Done	Ding et al. (2013)
	Photosensitive	Methoxyl-poly (ethylene glycol)-block-poly (lactide-co-2-methyl-2-carboxyl-propylene carbonate-ethanol amine)	Platinum (IV) prodrug (UVA-Pt2)	-	Done	Rong et al. (2014)
		Methoxy-poly (ethylene glycol)-block-poly (e-caprolactone)-block-poly-L-lysine, mPEG_114_-b-PCL_20_-PLL_10_	Cisplatin	Done	-	Hai et al. (2015)
		MMPs-specific PEGylated peptide	Doxorubicin	Done	Done	Lee et al. (2007)
Dendrimers	Ordinary	FA- and FI-modified {(Au^0^)_51.2_-G5·NH_2_} DENPs	-	Done	-	Shi et al. (2007)
		D4@3-As-DzSur with AS1411 as targeting ligands and anti-survivin DNAzyme as therapeutic agents	Doxorubicin	Done	Done	Gholami et al. (2017)
Polymersomes	Thermo-sensitive	Poly (N-isopropylacrylamide)-chitosan (PNIPAAm-CS)	Ocular drug	Done	Done	Cao et al. (2007)
		Poly (N-isopropylacrylamide-co-acrylamide-co-allylamine) (NIPA-AAm-AH) nan	Doxorubicin	Done	-	Rahimi et al. (2008)
	PH-sensitive	Poly (ethylene glycol)-polyester	Doxorubicin/Paclitaxe	Done	Done	Ahmed et al. (2006)
Hydrogel	Temperature-sensitive	Poly (N-isopropylacrylamide) gels/tailored nanoporous silica	Indomethacin	-	-	Shin et al. (2001)
		Hydroxyapatite-poly (N-isopropylacrylamide) (HAP–PNIPAAm)	Indomethacin	-	-	Shin et al. (2002)
	pH-sensitive	HP-β-CD/agarose-g-poly (MAA)	Capecitabine	Done	-	Rehman et al. (2021)
	Electroresponsive	Poly (methylacrylic acid) (PMAA)/pristine multiwalled carbon nanotubes	Radio-labelled sucrose	Done	-	Servant et al. (2013)
		(pMWNT)				
MOFs	Ordinary	ZrCl_4_/terephthalic acid	5-fluorouracil (5-FU)/dichloroacetate (DCA)/ibuprofen (IBU)/alendronate	Done	-	Lazaro et al. (2020)
			(AL)			
		Amino-triphenyldicarboxylic acid (amino-TPDC)/ZrCl_4_/amino-TPDC	Cisplatin/siRNAs	-	Done	He et al. (2014)
	Stimuli-sensitive	2-Methylimidazole/chloroauric acid/sodium palladium tetra-chloride (Na_2_PdCl_4_)/poly (vinyl pyrrolidone) (PVP, 30,000 W)/L-ascorbic acid (AA)	Doxorubicin	Done	-	Yang et al. (2017)

## 3 Conclusion and outlook

Polymeric NPs, as a group full of vigor, have excellent prospects and commercial value. The whole development of polymeric NPs is based on theory, experiment, and practical application. At present, there are more than 60 nano-drugs being approved for marketing in the world, and more than 200 are under clinical research, among which anti-tumor drugs are the main ones, including antiviral drugs, anti-inflammatory drugs, polypeptide protein drugs, RNA drugs, and drugs for diagnostic imaging. Compared with other dosage form, NPs have the advantages of improving drug stability—avoiding the drugs being rapidly destroyed or degraded, prolonging circulation time of drugs, enhancing penetrating and retention effects, specific binding with tumor cells and achieving targeted delivery, blocking angiogenesis of tumor tissues, and so on. Nanomedicine shows great potential in improving drug distribution and improving bioavailability. It is developing steadily and lively. However, challenges in the preparation of NP materials, improvement of their biological compatibility, the unknown mechanism of drug carrying, and the toxicity of polymeric NP materials *in vivo* are the problems remaining to be solved. Additionally, the pharmacokinetic of most NPs *in vivo* and the intracorporal processes of drugs are still not clear, which lead to the different results between clinical efficacy and preclinical studies. This is also one of the important reasons that the clinical development of nanomedicine has been restricted. Based on the promising application prospect of NPs, we remain committed to solving these problems. First, diversified synthesis methods need to be used. Different nanocarriers need to be synthesized and selected for different drug molecules. At present, the synthesis of nanocarrier drugs is mostly in liquid phase, including hydrothermal synthesis, template synthesis, interface synthesis, and so on. More syntheses with new techniques need to be explored. Second, most of the polymeric NPs cannot be used in clinics because of their toxicity. Although a large number of biocompatible polymeric NP materials have been used, the study of their toxicity is still limited by the imperfect analysis methods for the mechanism of drug carriers. Third, the mechanism of action of NPs *in vivo* is under improvement. The pharmacokinetic study of NPs should also flourish with the development of drug delivery systems. To obtain the transport rate between plasma drug concentration and tissue drug concentration by establishing a reasonable pharmacokinetic model has an important meaning for the design and optimization of NPs. To sum up, it can be predicted that polymeric NP carriers will be developed and widely applied with the further improvement of synthetic methods, analytical methods, and the basic experimental work of pharmacology. In addition, with the development of sensing, interconnection, and big data technologies, mobile medical system and miniaturization of drug delivery are also important tasks in the future.

## Glossary

NPs: nanoparticles
PEG: polyethylene glycol
EPR: enhanced permeability and retention effect
pH_E_: extracellular pH
PDEA: poly(N,N-diethylaminoethyl methacrylate)
PVP: poly(4- or 2-vinylpyridine)
PHis: poly(L-histidine)
PbAE: poly(β-amino ester)
pH_T_: pH of the transition state from insolubility to solubility
DOX: doxorubicin
PEGb-PDPA: poly(ethylene glycol)-block-poly(2-(diisopropylamino)ethyl methacrylate)
TPGS: d-a-tocopheryl polyethylene glycol 1000 succinate
GSH: glutathione
mPEG: methoxyl poly(ethylene glycol)
PZLL: poly(3-benzyloxycarbonyl-L-lysine)
PEG-b-PHPMA-LA: poly(ethylene glycol)-b-poly(N-2-hydroxypropyl methacrylamide)-lipoic acid
DTT: dithiothreitol
CPT: camptothecin
UVA: ultraviolet A
BU-PPG: uracil-end-capped oligomeric poly(propylene glycol)
BA-PPG: adenine-end-capped oligomeric poly(propylene glycol)
RES: reticuloendothelial system
LCL: long circulation liposomes
GM-1: gangliosides
PEGS: polyethylene glycol derivatives
PEI 1800-TA: polyethylenimine 1800-triamcinolone acetonide
CHEDLA: [(2-lactoylamido) ethylamino]formic acid cholesterol ester
STL: successive targeting liposomes
PE: phosphatidyl ethanolamine
DOPE: dioleoyl phosphatidyl ethanolamine
FA: folic acid
FI: fluorescein isothiocyanate
KB cells: a human epithelial carcinoma cell line
PAMAM-G7: dendritic polyamides-generation 7
PNIPAAm: poly(N-isopropylacrylamide)
LCST: lower critical solution temperature
PMPC-PDPA: poly (2-(methacryloyloxy)ethyl phosphorylcholine)-b-poly (2-(diisoprop ylamino) ethylm ethacrylate
PBD-PGA: di-block copolymer poly(butadiene)-b-poly(L-glutamic acid)
Lig: lignosulfonate
SWNCT: single-walled carbon nanotubes
MOFs: metal–organic frameworks
NMOFs: nanometer scale metal–organic frameworks
aMMTm: apatinib-loaded MOFs coated with MnO_2_ and decorated with membranes
5-FU uracil: 5-fluorouracil
Br-BODIPY: 1,3,5,7-tetramethyl-4,4-difluoro-8-bromomethyl-4-bora-3a,4a-diaza-s-indaeene
ES-CP: ethoxysueeinato-cisplatin
HMS: hollow mesoporous silica
AFM: atomic force microscopy
TEM: transmission electron microscopy.
